# Cytochrome *c* oxidase response to changes in cerebral oxygen delivery in the adult brain shows higher brain-specificity than haemoglobin^☆^

**DOI:** 10.1016/j.neuroimage.2013.05.070

**Published:** 2014-01-15

**Authors:** Christina Kolyva, Arnab Ghosh, Ilias Tachtsidis, David Highton, Chris E. Cooper, Martin Smith, Clare E. Elwell

**Affiliations:** a Dept. of Medical Physics and Bioengineering, University College London, London, UK; b Neurocritical Care Unit, University College Hospitals, London, UK; c Biological Sciences, University of Essex, Colchester, UK

**Keywords:** Cytochrome *c* oxidase, Hypoxia, Hyperoxia, Hypocapnia, Hypercapnia, Near-infrared spectroscopy

## Abstract

The redox state of cerebral mitochondrial cytochrome *c* oxidase monitored with near-infrared spectroscopy (Δ[oxCCO]) is a signal with strong potential as a non-invasive, bedside biomarker of cerebral metabolic status. We hypothesised that the higher mitochondrial density of brain compared to skin and skull would lead to evidence of brain-specificity of the Δ[oxCCO] signal when measured with a multi-distance near-infrared spectroscopy (NIRS) system. Measurements of Δ[oxCCO] as well as of concentration changes in oxygenated (Δ[HbO_2_]) and deoxygenated haemoglobin (Δ[HHb]) were taken at multiple source-detector distances during systemic hypoxia and hypocapnia (decrease in cerebral oxygen delivery), and hyperoxia and hypercapnia (increase in cerebral oxygen delivery) from 15 adult healthy volunteers. Increasing source-detector spacing is associated with increasing light penetration depth and thus higher sensitivity to cerebral changes. An increase in Δ[oxCCO] was observed during the challenges that increased cerebral oxygen delivery and the opposite was observed when cerebral oxygen delivery decreased. A consistent pattern of statistically significant increasing amplitude of the Δ[oxCCO] response with increasing light penetration depth was observed in all four challenges, a behaviour that was distinctly different from that of the haemoglobin chromophores, which did not show this statistically significant depth gradient. This depth-dependence of the Δ[oxCCO] signal corroborates the notion of higher concentrations of CCO being present in cerebral tissue compared to extracranial components and highlights the value of NIRS-derived Δ[oxCCO] as a brain-specific signal of cerebral metabolism, superior in this aspect to haemoglobin.

## Highlights

• NIRS was used to measure oxidised cytochrome *c* oxidase (Δ[oxCCO]) in healthy brain.
• Δ[oxCCO] changed in the same direction as changes in cerebral oxygen delivery.
• Magnitude of Δ[oxCCO] response increased with increasing light penetration depth.
• Corresponding haemoglobin changes showed no dependence on light penetration depth.
• NIRS-measured Δ[oxCCO] has higher brain specificity than haemoglobin.

## Introduction

Transcranial near-infrared spectroscopy (NIRS) provides a measure of cerebral oxygen delivery by monitoring concentration changes in oxygenated (Δ[HbO_2_]) and deoxygenated haemoglobin (Δ[HHb]), non-invasively. A third spectral signal is present (Tisdall et al., 2008a), consistent with the features of mitochondrial cytochrome *c* oxidase (CCO) (Jöbsis, 1977). CCO is the terminal electron acceptor in the mitochondrial respiratory chain and, being responsible for over 95% of oxygen metabolism, it is instrumental in aerobic ATP synthesis (Richter and Ludwig, 2003). Since in the short term the total concentration of CCO remains constant, concentration changes of oxidised cytochrome *c* oxidase monitored with NIRS (Δ[oxCCO]) represent changes in the CCO redox state, which reflects the balance between cerebral energy supply and demand (Smith, 2011). Thus, Δ[oxCCO] is an appealing target for the bedside assessment of regional cerebral metabolic status and oxygen utilisation, and provides information complementary to Δ[HbO_2_] and Δ[HHb], which only reflect intravascular oxygenation. The information is also complementary to cerebral oximetry, which delivers a measure of absolute cerebral haemoglobin oxygen saturation based on the technique of spatially resolved spectroscopy (SRS). Regardless of the high sensitivity and specificity of cerebral oximetry to intracerebral changes (Al-Rawi et al., 2001), it is still affected by extracerebral changes (Davie and Grocott, 2012) and so far it has not been successful in providing a robust clinical marker of sufficient cerebral oxygen delivery and tissue status (Boas and Franceschini, 2011; Ghosh et al., 2012).

Despite its potential as a non-invasive bedside marker of cerebral cellular oxygen metabolism, there is still debate about the use of Δ[oxCCO] outside research clinical settings, primarily due to technical complexities associated with this measurement in the adult brain, in the presence of significantly higher concentrations of haemoglobin. The possible interference of changes in optical scattering with the NIRS recordings and the insufficient chromophore separation by the algorithm used to convert optical density into concentration changes are the most notable challenges (Cooper and Springett, 1997; Cooper et al., 1999). A hybrid optical spectrometer (pHOS) and accompanying algorithm designed to address the above issues and provide robust Δ[oxCCO] data, have recently been developed by our group (Kolyva et al., 2012). In addition, the pHOS has the capacity for measurements at multiple source-detector distances (and therefore at multiple depths), a technological advance that enables for the first time multi-distance Δ[oxCCO] recordings in vivo in adults. These recordings may contribute considerably to the interpretation of the Δ[oxCCO] signal, by determining if there is a distance/depth-dependent response of Δ[oxCCO] in the adult head, an expectation based on the higher mitochondrial density of the brain compared to tissues with lower metabolic rates, such as skin and skull (Kakihana et al., 2008; Smith and Elwell, 2009; Tisdall et al., 2007).

The aim of the present study was to investigate the multi-depth response of Δ[oxCCO] to global changes in cerebral oxygen delivery secondary to systemic hypoxia, hyperoxia, hypocapnia and hypercapnia in the healthy adult brain. To enable the more thorough monitoring of the physiological mechanisms activated in the brain during oxygen delivery manipulation, cerebral blood flow velocity and absolute tissue oxygen saturation were studied simultaneously with Δ[HbO_2_], Δ[HHb] and Δ[oxCCO]. We hypothesised that Δ[oxCCO] would show an incremental response with increasing source-detector separation, mirroring potential differences in the extra- and intracranial distribution of this chromophore, and reinforcing confidence in the use of this signal as a brain-specific biomarker of cerebral metabolic status.

## Methods

This study was approved by the local ethics committee and all subjects provided written informed consent.

### Monitoring

#### NIRS monitoring

A hybrid spectroscopy device (pHOS) described in more detail elsewhere, was used to obtain multi-distance near-infrared measurements (Kolyva et al., 2012; Tachtsidis et al., 2010). The pHOS combines frequency domain (FD) and broadband (BB) components and can measure light absorption and scattering at discrete wavelengths (690, 750, 790 and 850 nm), together with broadband light attenuation in the range 504–1068 nm. Each of the two pHOS optodes incorporates an FD channel (source-detector spacing 3.0 and 3.5 cm) and BB channel (source-detector spacing 2.0, 2.5, 3.0 and 3.5 cm). One sampling cycle of the pHOS lasts 3.2 s, and BB and FD measurements are made sequentially. A single optode was placed on the forehead in the mid-pupillary line, high enough to avoid the frontal sinuses.

#### Other physiological monitoring

Other monitoring included beat-to-beat pulse oximetry (Oxypleth, NovaMetrix, MA), continuous non-invasive arterial blood pressure (PortaPres, Finapres Medical Systems, The Netherlands), electrocardiography (IntelliVue MP50, Philips Healthcare, The Netherlands), capnography (CO_2_SMO, NovaMetrix) and inspired/expired oxygen partial pressure (IntelliVue Anaesthetic Gas Module, Philips Healthcare). Transcranial Doppler (TCD) ultrasonography was used to measure middle cerebral artery flow velocity ipsilateral to the pHOS optode, as a surrogate of cerebral blood flow (DWL Doppler Box, Compumedics, Germany).

### Protocols

Cerebral oxygen delivery was manipulated through four separate systemic physiological challenges: hypoxia, hyperoxia, hypocapnia and hypercapnia.

**Fig. 1:**
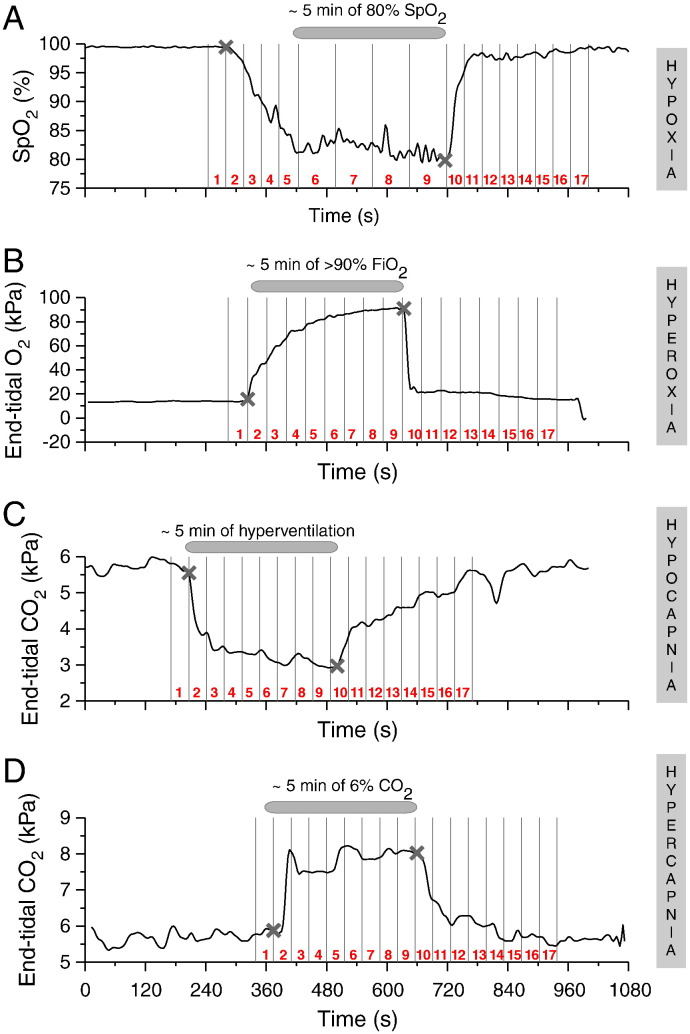
Examples of individual data illustrating the protocol for hypoxia (A), hyperoxia (B), hypocapnia (C) and hypercapnia (D), with the physiological signal most pertinent to the challenge plotted in each graph. The challenge period (denoted with two ‘x’ marks), is the interval during which the subject was not breathing room air (A, B, D) or was hyperventilating (C). Time interval 1 corresponds to baseline, intervals 2–9 cover the challenge period and intervals 10–17 comprise the period after the end of the challenge, with explanation on how these intervals were determined, provided in the text.

Each study protocol was commenced with 5 min of baseline recording (air inhalation). Subsequently, hyperoxia was instigated by increasing the inspired fraction of oxygen (FiO_2_) to > 90%. Analogously, hypercapnia was induced by the addition of 6% CO_2_ to the inspired gas mix, targeting an increase of ~ 2 kPa in end-tidal partial pressure of CO_2_ (EtCO_2_). Hypocapnia was achieved by instructing the subjects to hyperventilate; verbal feedback was provided to them for guidance, in order to reach and maintain a ~ 2 kPa reduction in EtCO_2_. Each of these manipulations lasted 5 min (‘*challenge*’ period) (Figs. 1B–D). The protocols were then concluded with a further 5-minute period of baseline recording. In the hyperoxia/hypercapnia protocols, this second period of baseline commenced after the return of end-tidal O_2_/CO_2_ partial pressures to their pre-challenge levels, once inhalation of room air had been resumed. In the hypocapnia protocol, the second period of baseline recording directly followed the cessation of hyperventilation.

The hypoxia protocol differed from the others to account for physiological variability in the speed of haemoglobin desaturation during inhalation of a hypoxic gas mix. After the initial 5-minute period of baseline recording, hypoxaemia was induced through the delivery of a hypoxic gas mix: the initial FiO_2_ of 8% was titrated to achieve a reduction in arterial oxygen saturation (SpO_2_) to 80% (‘*induction’* period). Once this was accomplished, SpO_2_ was sustained at 80% for 5 min (‘*plateau*’ period), before returning the inspired gas to room air (Fig. 1A). After return of end-tidal O_2_ back to baseline levels, the recording was continued for a further 5-minute period of baseline recording.

For hypoxaemia, hyperoxia and hypercapnia, a sequential gas delivery circuit was used to maintain a constant EtCO_2_ despite changes in minute volume (Slessarev et al., 2007). For hypocapnia, the subjects were instructed to hyperventilate through a mouthpiece incorporating the sensors for capnography and tidal oximetry.

### Data analysis

#### NIRS algorithms

##### Differential spectroscopy

Data analysis was performed in MATLAB (version R2010b, MathWorks, Natick, MA). Based on the modified Beer–Lambert law, Δ[HbO_2_]), Δ[HHb]) and Δ[oxCCO] were determined from the measured changes in broadband light attenuation using the UCL*n* algorithm, a least-squares fitting technique (Kolyva et al., 2012; Matcher et al., 1995). The wavelength range used for the fitting was 780–900 nm and the wavelength dependence of differential pathlength factor (DPF) (Essenpreis et al., 1993), demonstrated as a decrease in DPF with increasing wavelength, was incorporated in the calculations, which were carried out individually for the four detectors. DPF was assumed to be equal to 6.26 for the two detectors proximal to the BB light source (source-detector spacing 2.0 and 2.5 cm) (Duncan et al., 1995) and was calculated for the two distal detectors (source-detector spacing 3.0 and 3.5 cm) using an initial FD measurement at 790 nm (Fantini et al., 1999). Changes in total haemoglobin concentration were defined as Δ[HbT] =Δ[HbO_2_] + Δ[HHb] and in haemoglobin difference as Δ[Hbdiff] =Δ[HbO_2_] − Δ[HHb]. The absorption (μ_a_) and reduced scattering (μ_s_′) coefficients were quantified from the FD measurements.

##### Spatially resolved spectroscopy

The tissue oxygenation index (TOI) was calculated using the SRS methodology, through which the slope of BB light attenuation within the range 740–900 nm was derived (Suzuki et al., 1999).

Linear regression was performed between the measured attenuations (A) of the four detectors and the corresponding source-detector distances (ρ) (assuming that the latter were the nominal distances described in the NIRS monitoring section), yielding an attenuation slope (∂A / ∂ρ). At every sampling point, this calculation was repeated for every wavelength (λ) within the range 740–900 nm. This in turn enabled the derivation of a relative absorption coefficient spectrum at every sampling point, according to the equation (Suzuki et al., 1999):

(1) $${\mathrm{k\mu}}_{a}\left(\lambda \right)=\frac{1}{3\left(1-\mathrm{h\lambda}\right)}{\left(\ln10\cdot \frac{\partial A\left(\lambda \right)}{\partial \rho }-\frac{2}{\bar{\rho }}\right)}^{2}\text{.}$$

The above equation originates from the diffusion approximation, and (1 − hλ) is a correcting factor that accounts for the wavelength-dependency of light scattering (Matcher et al., 1997), while $\bar{\rho }$ is the average source-detector spacing of the four detectors and k is an unknown constant. Assuming 70% water concentration in the tissue interrogated by the BB light, the contribution of the water absorption spectrum was subtracted from the kμ_a_ spectrum and the resulting spectrum was used for the calculation of the relative concentration of oxygenated (k[HbO_2_]) and deoxygenated haemoglobin (k[HHb]). TOI was derived as the ratio k[HbO_2_] / (k[HbO_2_]) + k[HHb]).

All calculated concentrations and TOI were linearly detrended for the removal of baseline drifts and low-pass filtered with a 5th order Butterworth filter (cut-off frequency 0.08 Hz).

#### Definition of average time points

The measured O_2_ and CO_2_ partial pressure waveforms yielded inspired and end-tidal gas partial pressures, from which 3.2-second averages were computed together with 3.2-second averages for SpO_2_, arterial blood pressure (yielding mean arterial blood pressure, ‘*MBP’*) and TCD-measured velocity envelope (yielding mean flow velocity in the middle cerebral artery, ‘*Vmca*’), while heart rate (HR) was interpolated every 3.2 s. After this additional discretization, these data were combined with the optical data into a single data set per subject.

To enable data averaging across subjects despite the variation in the timing of their physiological responses, the recorded data for each volunteer and protocol were split into 17 phases, which were subsequently averaged over subjects to yield grand averages for hypoxia, hyperoxia, hypocapnia and hypercapnia.

For the hyperoxia, hypercapnia and hypocapnia protocols the beginning and end of the 5-minute challenge period was identified from the measured partial pressures of O_2_ or CO_2_ as appropriate (denoted with two ‘x’ marks in the examples shown in Figs. 1B–D). This period was optimally split into 8 equal phases, which represented time intervals ‘2–9’, while time interval ‘1’ corresponded to the baseline immediately prior to the onset of the challenge, and time intervals ‘10–17’ covered the period after the end of the challenge; all intervals ‘1–17’ had equal duration.

For the hypoxia protocol, variability between subjects in the time taken to achieve the targeted SpO_2_ of 80%, and thus variability in the duration of the induction period, mandated a different approach. The onset of the induction period and the end of the plateau period were identified from the measured partial pressure of O_2_ (denoted with two ‘x’ marks in the example shown in Fig. 1A); the onset of the plateau period was identified from the measured SpO_2_. The induction period was optimally split into 4 equal phases (time intervals ‘2–5’) and the plateau period to another 4 equal phases (time intervals ‘6–9’). Time interval ‘1’ corresponded to the baseline immediately prior to the onset of the induction period and time intervals ‘10–17’ covered the period after the end of the plateau, these intervals mirroring in duration interval ‘2’.

For each of the 17 time intervals, representative optical and systemic data were derived by averaging the last 9 ∗ 3.2 s of data from each of the corresponding 17 data segments.

### Statistical analysis

Statistical analysis was carried out in SPSS (PASW Statistics for Windows, version 18.0, SPSS Inc, Chicago, IL). The data were assessed for normality through Q–Q plots. Two types of comparisons were performed thereafter in the group data and repeated for each of the four challenges.

Firstly, for all parameters of interest, repeated measures ANOVA tests with Greenhouse–Geisser correction were performed to determine whether the group means overall changed significantly between time points 1–17. Post hoc tests with Bonferroni corrections determined exactly at which time points 2–17 there was a statistically significant change compared to point 1. The duration of all individual recordings was sufficiently long to extend beyond the end of the challenge and subsequent return to baseline. However, for a few subjects the period recorded after the end of one or more challenges was not long enough to acquire the full set of time intervals 11–17. Consequently, for hypoxia we conducted the statistical tests only between time intervals 1–10, for hyperoxia between 1–14, for hypocapnia between 1–11 and for hypercapnia between 1–15, for which data were available from all 15 volunteers.

The second type of comparison involved, for all BB-derived chromophore concentrations, repeated measures ANOVA tests at each time point, to determine whether the concentration changes measured by the four detectors were overall statistically significantly different between detectors. Subsequently, post hoc tests with Bonferroni corrections determined exactly which detectors showed a statistically significant difference with respect to their neighbouring detectors.

Average data are expressed as mean ± standard error of the mean (SEM) and statistical significance was assumed at P < 0.05.

## Results

Summary demographic data, separately for the four challenges, are given in Table 1. The hypoxia, hyperoxia, hypocapnia and hypercapnia protocols were each completed by 15 healthy adult volunteers, with a total of 23 subjects participating in the study. The table also includes group data at baseline (time point 1) and at the end of the challenge (time point 9) for a number of measured systemic parameters. During the hypoxia challenge there was an 18% decrease in SpO_2_ (P < 0.0001) between points 1 and 9. The hyperoxia challenge was accompanied by an increase in end-tidal partial pressure of O_2_ from 14.3 ± 0.3 kPa at point 1, to 89.2 ± 1.4 kPa at point 9 (P < 0.0001). The hypocapnia challenge induced a considerable decrease of 43% in EtCO_2_ (P < 0.0001) between points 1 and 9. Conversely, hypercapnia resulted in a 45% increase in EtCO_2_ (P < 0.0001) between the same time points.

**Fig. 2:**
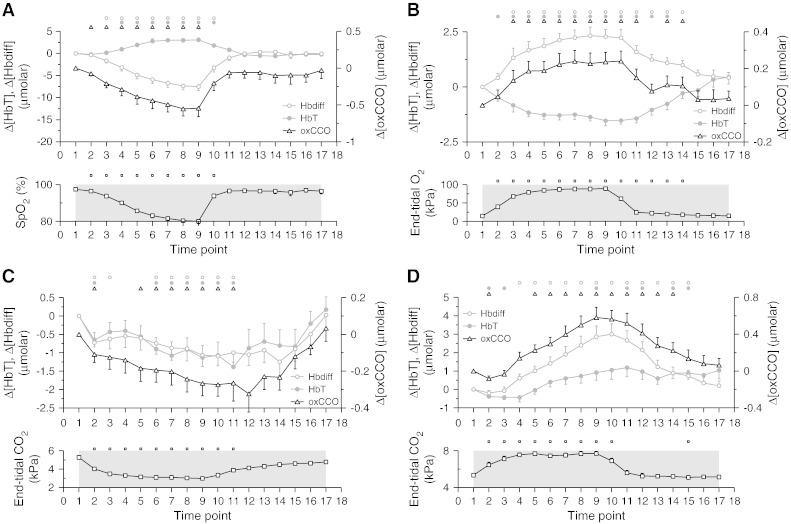
Grand averages of the time courses of Δ[Hbdiff], Δ[HbT] and Δ[oxCCO] measured by the detector distal to the light source (source-detector separation of 3.5 cm), over the 15 volunteers that participated in the hypoxia (A), hyperoxia (B), hypocapnia (C) and hypercapnia (D) protocols. The corresponding traces of arterial oxygen saturation (SpO_2_) (A), end-tidal O_2_ partial pressure (B) and end-tidal partial CO_2_ pressure (C and D) are also provided for reference. The small symbols on top of each plot indicate statistical significance with respect to time point 1 (P < 0.05) for the parameters plotted in matching symbols and the error bars represent the standard error of the mean. For reasons explained in the text, we performed the statistical tests only for time intervals 1–10 for hypoxia, 1–14 for hyperoxia, 1–11 for hypocapnia and 1–15 for hypercapnia.

Group grand averages of the time courses of Δ[oxCCO], Δ[HbT] and Δ[Hbdiff] as measured during hypoxia and recovery by the distal detector of the optical array (3.5 cm source-detector spacing) are presented in Fig. 2A. During hypoxia, there was a decrease in Δ[oxCCO], accompanied by an increase in Δ[HbT] and decrease in Δ[Hbdiff] (P < 0.0001 for all). Fig. 2B displays the group time courses of Δ[oxCCO], Δ[HbT] and Δ[Hbdiff] registered by the same detector during hyperoxia and recovery; an increase in Δ[oxCCO] (P < 0.0001), was accompanied by a decrease in Δ[HbT] (P < 0.001) and an increase in Δ[Hbdiff] (P < 0.0001) during this challenge. The corresponding traces during hypocapnia and recovery are shown in Fig. 2C, with a decrease in Δ[oxCCO] (P < 0.005) observed in the presence of a decrease in both Δ[HbT] (P < 0.005) and Δ[Hbdiff] (P < 0.05). In Fig. 2D, which depicts group data from the distal detector during hypercapnia and recovery, an increase in Δ[oxCCO] (P < 0.0001) was associated with an increase in both Δ[HbT] (P < 0.005) and Δ[Hbdiff] (P < 0.0001).

**Fig. 3:**
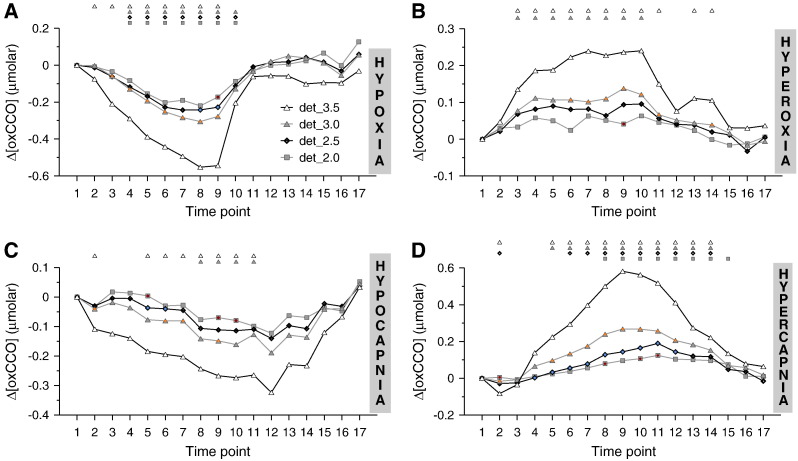
Group data of the time courses of Δ[oxCCO] measured by the detectors 2.0, 2.5, 3.0 and 3.5 cm away from the light source (denoted, respectively, as det_2.0, det_2.5, det_3.0, and det_3.5). The averages are based on the 15 volunteers that participated (A) in the hypoxia challenge, (B) in the hyperoxia challenge, (C) in the hypocapnia challenge and (D) in the hypercapnia challenge. The small symbols on top of each plot indicate statistical significance with respect to time point 1 (P < 0.05) for the parameters plotted in matching symbols, while the coloured circles indicate statistically significant differences (P < 0.05) between detectors (yellow: det_3.0 from det_3.5; blue: det_ 2.5 from det_3.0; red: det_2.0 from det_2.5).

Fig. 3 provides a combined view of the Δ[oxCCO] response to hypoxia, hyperoxia, hypocapnia and hypercapnia from all four detectors (denoted as ‘*det_2.0*’, ‘*det_2.5*’, ‘*det_3.0*’, and ‘*det_3.5*’, for source-detector spacing of 2.0, 2.5, 3.0 and 3.5 cm, respectively). In terms of directional changes, within each challenge the Δ[oxCCO] response was uniform between all detectors, with Δ[oxCCO] consistently decreasing during hypoxia (P < 0.0001 for all detectors) and hypocapnia (det_3.5: P < 0.005, det_3.0: P < 0.05, det_2.5 and det_2.0: P = NS) and increasing during hyperoxia (det_3.5: P < 0.0001, det_3.0: P < 0.05, det_2.5 and det_2.0: P = NS) and hypercapnia (det_3.5 and det_3.0: P < 0.0001, det_2.5: P < 0.0005, det_2.0: P < 0.05). For all challenges the magnitude of the Δ[oxCCO] response gradually increased from the proximal to the distal detectors as shown in Fig. 3, with differences between neighbouring detectors reaching statistical significance at several time points.

**Fig. 4:**
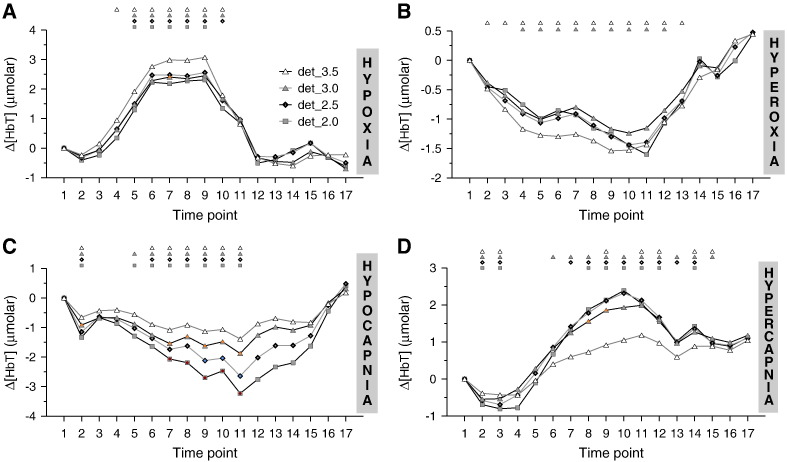
Group data of the time courses of Δ[HbT] measured by the detectors 2.0, 2.5, 3.0 and 3.5 cm away from the light source (denoted, respectively, as det_2.0, det_2.5, det_3.0, and det_3.5). The averages are based on the 15 volunteers that participated (A) in the hypoxia challenge, (B) in the hyperoxia challenge, (C) in the hypocapnia challenge and (D) in the hypercapnia challenge. The small symbols on top of each plot indicate statistical significance with respect to time point 1 (P < 0.05) for the parameters plotted in matching symbols, while the coloured circles indicate statistically significant differences (P < 0.05) between detectors (yellow: det_3.0 from det_3.5; blue: det_ 2.5 from det_3.0; red: det_2.0 from det_2.5).

For each challenge the directional response of Δ[Hbdiff] and Δ[HbT] (Fig. 4) was also uniform between detectors, following the same patterns shown in Fig. 2 for det_3.5. The magnitude of the Δ[Hbdiff] and Δ[HbT] response did not exhibit a gradient response between detectors as Δ[oxCCO] did, but was instead similar in all detectors, with the exception of Δ[HbT] in hypocapnia and Δ[Hbdiff] in hypoxia, which both showed a gradually decreasing response from the proximal to the distal detectors, with differences between adjacent detectors being statistically significant at a few time points.

Table 2 provides the average pathlength data used for the chromophore concentration calculations reported above.

**Fig. 5:**
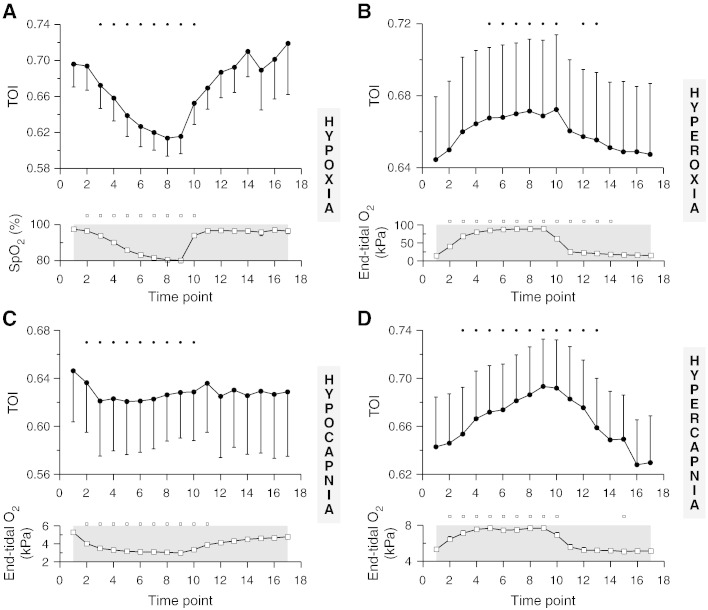
Group data of the time course of TOI, for hypoxia (A), hyperoxia (B), hypocapnia (C) and hypercapnia (D). The corresponding traces of SpO_2_ (A), end-tidal partial O_2_ pressure (B) and end-tidal partial CO_2_ pressure (C and D) are also provided for reference. The small symbols on top of each plot indicate statistical significance with respect to time point 1 (P < 0.05) and the error bars represent the standard error of the mean.

Fig. 5 presents grand averages of the TOI response to the hypoxia, hyperoxia, hypocapnia and hypercapnia challenges. There was a statistically significant increase in TOI during hyperoxia (P < 0.05) and hypercapnia (P < 0.0005) and a statistically significant decrease during hypoxia (P < 0.0001) and hypocapnia (P < 0.05). There was no statistically significant change in Vmca during hyperoxia and an increase of almost 15% during hypoxia (P < 0.0005). The changes in Vmca were more pronounced during the CO_2_ challenges, with a considerable decrease of 30% observed during hypocapnia and a dramatic increase of approximately 60% observed during hypercapnia (P < 0.0001 for both) (Supplementary Fig. 1).

No changes in μ_s_′ (P = NS for all wavelengths) were measured simultaneously with the chromophore concentration changes, during any of the four challenges.

## Discussion

### Summary of findings

Four different experimental paradigms of cerebral oxygen delivery manipulation in healthy adults (systemic hypoxia, hyperoxia, hypocapnia and hypercapnia) were investigated. An increase in Δ[oxCCO] was observed during the challenges that increased cerebral oxygen delivery and, analogously, a decrease in Δ[oxCCO] took place when cerebral oxygen delivery decreased. A consistent pattern of gradually increasing amplitude of the Δ[oxCCO] response with increasing light penetration depth was observed in all four paradigms, with the direction of the Δ[oxCCO] response remaining uniform at different penetration depths within the same challenge. The response of Δ[oxCCO] was distinctly different from the Δ[HbT] and Δ[Hbdiff] responses, both in terms of directional changes and in terms of demonstrating a penetration depth gradient, the latter a behaviour that was seldom observed in the haemoglobin signals. Regional cerebral tissue oxygen saturation, as indicated by TOI, was increased during hyperoxia and hypercapnia and decreased during hypoxia and hypocapnia, showing for each challenge the same trend as Δ[oxCCO].

### Comparison with previous studies

#### Differential spectroscopy

Because of discrepancies that can be introduced by the use of different instruments, algorithms (Matcher et al., 1995), experimental paradigms and subject populations, a quantitative comparison of the chromophore concentration changes measured in the current study will be attempted only with other BB spectroscopy studies in the healthy adult head that also used the UCL*n* algorithm and similar source-detector separations. In this context, our results are broadly in agreement with recently published studies that were conducted with a single source-detector spacing of 3.5 cm, during manipulation of cerebral oxygen delivery (Tachtsidis et al., 2009; Tisdall et al., 2007). In one such study, a decrease in cerebral oxygen delivery, induced by a gradual fall in SpO_2_ from 100% to 80% and immediate return to normoxia, was followed by a 0.24 μM reduction in Δ[oxCCO] (median value) (Tisdall et al., 2007), while the corresponding decrease measured in our volunteer population during hypoxia at time point 9 was 0.46 μM. In another study, increased cerebral oxygen delivery, via increased cerebral blood flow induced by a rise of 1.5 kPa in EtCO_2_, resulted in a 0.25 μM rise in Δ[oxCCO] (mean value) (Tachtsidis et al., 2009). Fig. 2D demonstrates that the changes observed in our study during hypercapnia, triggered by a rise in EtCO_2_ of 2.4 kPa (Table 1), were not dissimilar. Finally, previously published BB data collected during a second protocol for cerebral oxygen delivery augmentation, through an increase in arterial oxygen content, revealed a rise of 0.09 μM in Δ[oxCCO] (mean value) (Tachtsidis et al., 2009). Following an identical hyperoxia protocol, the present study yielded similar findings, illustrated in Fig. 2B. We note that there are some differences in the amplitude of the responses measured in the current study compared to the above literature. We believe that these dissimilarities can be primarily accounted for by the fact that Tisdall et al. (2007) and Tachtsidis et al. (2009) used second differential analysis of the 740 nm water feature to calculate optical pathlength, whereas the present study followed a different method, described in detail in the Differential spectroscopy section in Methods.

Solely qualitative comparisons, for reasons explained in the previous paragraph, are on the other hand feasible with several other studies that span a wide range of methodologies. A study conducted with a commercial 4-wavelength system (NIRO 500, Hamamatsu Photonics) during a 2.1 kPa drop in EtCO_2_ caused by hyperventilation, reported a minor but not statistically significant decrease in Δ[oxCCO] in the healthy adult brain (Grubhofer et al., 1999). This finding, established with a 6.0 cm source-detector spacing, broadly concurs with our hypocapnia data (Fig. 2C) that were collected under a similar EtCO_2_ decrease (Table 1). In adult patients with traumatic brain injury, studies both with the pHOS and with BB spectroscopy at 3.5 cm source-detector spacing, showed an increase in Δ[oxCCO] during normobaric hyperoxia (100% FiO_2_), concurring with the findings of the present study (Ghosh et al., 2013; Tisdall et al., 2008b). A clinical study on newborn preterm infants with a 6-wavelength system (NIRO 1000, Hamamatsu Photonics) operating in transmission mode, was able to show that an increase in arterial carbon dioxide tension was accompanied by an increase in Δ[oxCCO], supporting the outcome of our hypercapnia protocol (Edwards et al., 1991). The same clinical study reported however no change in Δ[oxCCO] during changes in arterial oxygen saturation.

Some (Cooper et al., 1997; Hoshi et al., 1997) but not all (Newman et al., 2000; Quaresima et al., 1998) animal studies suggest that CCO is fully oxidised in normoxia and thus cannot increase from baseline. However, in our studies we observed a consistent increase in the Δ[oxCCO] signal in hypercapnia and hyperoxia, suggesting that at normoxic normocapnia CCO is indeed not fully oxidised. Our studies were carried out in healthy awake humans rather than anaesthetised animals and it is highly likely that anaesthesia will have a significant impact on the relationship between oxygen supply and demand, and CCO oxidation. The idea of CCO not being fully oxidised at baseline is consistent with other groups who observe CCO oxidations using similar awake adult subject groups as ours with different experimental paradigms (Heekeren et al., 1999).

#### SRS

The measured baseline TOI values (time point 1), fall well within the normal range of 60–75% and the wide intra-subject variability indicated by the substantial error bars in Fig. 5 is also within normal range (Thavasothy et al., 2002). The observed TOI changes are also in very good agreement with previously published findings in the healthy adult brain during hypoxia, hyperoxia, hypocapnia and hypercapnia using a commercial 3-wavelength system which performs SRS based on two detectors (NIRO 300, Hamamatsu Photonics) (Tisdall et al., 2009). This earlier study reports a 7.1% decrease in TOI during hypoxia, 2.3% increase during hyperoxia, 2.1% decrease during hypocapnia and 2.6% increase during hypercapnia (median values) (Tisdall et al., 2009). Despite slight differences in the experimental protocols and the evident differences between the NIRO technology and the way in which TOI was derived in the present study, based on multi-wavelength measurements from four detectors, the TOI changes measured during the various challenges were markedly similar; at time point 9 we observed a 6.0% decrease in TOI during hypoxia, 3.2% increase during hyperoxia, 1.7% decrease during hypocapnia and 5.7% increase during hypercapnia (median values). Even though broadband-derived TOI has been measured before (in skin) (Liu et al., 2011), to our knowledge this is the first study to implement this technology in the adult head, and the specific combination of wavelength range and number of detectors is novel.

### Source-detector spacing and depth-dependence of NIRS signals

Theoretical modelling in the adult head (Firbank et al., 1998; Okada et al., 1997) suggests that for short source-detector spacing, the sampling volume investigated is almost entirely confined to non-cerebral tissue, such as scalp and skull. As the distance is increased, a progressively greater proportion of the light will pass through the cerebral cortex. Thus, during multi-distance NIRS recordings, intracerebral changes in chromophore content are going to produce a larger signal in the detectors more distal to the light source; this signal will progressively diminish in the proximal detectors. Conversely, the proximal detectors will have higher sensitivity to superficial changes. These theoretical predictions are in agreement with experimental studies in the adult head, where the differential sensitivity of detectors with short and long spacing was investigated through experimental protocols intended to selectively affect either scalp or cerebral oxygenation (Germon et al., 1998, 1999).

The above remarks refer to the *primary* predisposition of detectors with long spacing to trace intracranial changes, without however precluding the possibility of extracranial events contributing to the signal as well. As shown in a study during carotid endarterectomy, even with a relatively wide source-detector spacing of 6 cm, there was a joint contribution to the NIRS recordings from extracerebral and intracerebral vascular beds supplied, respectively, by the external and internal carotid arteries (Lam et al., 1997). The relative contribution of superficial and cerebral changes will not only depend on source-detector spacing, but also on the head and cortical anatomy of each subject (Haeussinger et al., 2011) and instrumentation specifications (for example wavelengths and intensity of the light source). It is therefore not possible to provide a quantitative estimation of these relative weightings for the specific source-detector spacings used in the present study. Only a qualitative statement about the intracranial contribution being expected to become progressively larger from det_2.0 to det_3.5 would be prudent.

### Depth-dependence of Δ[oxCCO]

These first multi-depth Δ[oxCCO] measurements during global changes in cerebral oxygen delivery have revealed a gradual increase in the amplitude of the Δ[oxCCO] response with increasing penetration depth (Fig. 3). The penetration depth gradient was in fact robust enough for statistically significant differences to be discernible not merely between det_2.0 and det_3.5, but even between adjacent detectors. As a secondary remark, it is noted that det_3.5 appears in some challenges to also register *earlier* than the other detectors changes in Δ[oxCCO], besides *larger* changes in magnitude. These differences could indicate that anatomically and physiologically distinct tissues were being monitored. Such spatial differentiation of the Δ[oxCCO] response most likely results from differences in the concentration distribution of CCO in the adult head. It has been suggested that due to higher mitochondrial density present in the brain than in tissues with lower metabolic rates, higher concentrations of CCO can be expected to be present in the brain than in the skin and the skull (Kakihana et al., 2008; Smith and Elwell, 2009; Tisdall et al., 2007). Direct evidence for the existence of such a spatial distribution has been provided for cytochrome *c*, with a study in rats reporting a proportion of 1:10 between skin and brain cortex cytochrome *c* contents (Drabkin, 1951). Apart from the present study, other evidence for the spatial distribution of CCO in the adult head is not available, primarily because it was not possible to make the necessary measurements in vivo.

It is possible that at long source-detector separations the amplitude of the responses shown in Fig. 3 will stop increasing further with increasing penetration depth and present a plateau instead, indicating that beyond a certain penetration depth the interrogated tissue has a fairly uniform CCO content. However, we believe that 3.5 cm source-detector spacing is insufficient to observe this plateau. As mentioned in the previous section, the distal detectors have higher sensitivity to intracerebral changes, but that is not to say that they are exclusively interrogating cerebral tissue, or that there is no difference in the amount of cerebral tissue included within the sampling volume of det_3.0 and det_3.5. Monte-Carlo modelling of light propagation suggests that the contribution of the brain to the overall NIRS signals increases beyond 3.5 cm (Okada et al., 1997), although in practice there is a trade-off to be made between this increased brain-specificity and a decreased signal intensity. This concern about reduced signal-to-noise ratio prompted our decision to limit source-detector separation to 3.5 cm in the studies presented here, but in future studies we would consider using additional longer source-detector separations worthwhile.

When the tissue interrogated with NIRS is the adult head, there are concerns about the risk of extracerebral tissue interfering with the intracranial signals. Solutions to circumvent this problem are constantly being sought, and currently include inter-channel subtraction using additional NIRS measurements from short source-detector separations (sensitive mainly to superficial layers) to remove the extracerebral interference present in longer source-detector separations (Gagnon et al., 2012), new analytical methods and post-processing algorithms, development of advanced spectroscopic methods (SRS, time-resolved systems; Aletti et al., 2012) and hybrid technologies (Gunadi and Leung, 2011).

Our results suggest that an alternative approach to this problem could be to target a signal that is brain-specific. The presence of the superficial layers adds more complexity to the extraction of the intracranial Δ[HHb] and Δ[HbO_2_] signals rather than Δ[oxCCO] because of the different spatial distribution of these chromophores, with CCO expressed predominantly in cerebral tissue and haemoglobin present both in cerebral and extracerebral tissues. Essentially, haemoglobin is both an interfering signal (because extracerebral haemoglobin concentration variations caused by physiological changes in the scalp can interfere with the intracranial signals) *as well as* a signal of interest (intracerebral haemoglobin), while CCO is less intricately coupled to scalp perfusion because it is present in considerably higher concentrations in cerebral than in extracerebral tissue. The depth-dependence of the Δ[oxCCO] signal identified in this study corroborates the notion of higher concentrations of CCO being present in cerebral tissue compared to extracranial components; it thereby highlights the value of NIRS-derived Δ[oxCCO] as a brain-specific signal that is less prone to extracerebral contamination than the haemoglobin signals and fortifies its clinical appeal as a non-invasive marker of regional cerebral metabolic status.

### Comparison of brain-specificity of Δ[oxCCO] to Δ[HbT] and Δ[Hbdiff]

All Δ[Hbdiff] and Δ[HbT] responses were in agreement with what would be physiologically expected, based on the systemic changes induced by the different protocols for cerebral oxygen delivery manipulation. Within the same challenge, the directional responses of Δ[Hbdiff] and Δ[HbT] were consistent across different source-detector separations, while the magnitude of these responses remained largely unchanged between detectors (Fig. 4). This uniformity in the magnitude of the responses, in contrast to the gradient response observed in the Δ[oxCCO] signal obtained from the *same* BB measurements, was indicative of a weakness of the haemoglobin signals to exhibit brain-specificity.

This difference was not merely an effect observed in the group responses, as we were able to substantiate with the following supplementary analysis. For each challenge, the 15 data sets from which the grand averages were derived, were individually inspected and the time point 1–17 at which the maximum Δ[oxCCO] response occurred (according to det_3.5), was noted. Subsequently, in a total of 60 data sets, the Δ[oxCCO], Δ[Hbdiff] and Δ[HbT] time courses were each graded: a score of 3, 2, 1 or 0 was assigned if, respectively, 3, 2, 1 or none detector pairs (det_3.5 and det_3.0, det_3.0 and det_2.5, det_2.5 and det_2.0) showed a larger response in the distal detector of the pair, at the time point noted above. For each challenge the scores for the 15 data sets were summed and the total scores of ‘brain-specificity’, presented as percentages of the maximum possible score, are provided in Table 3. As shown in the table, in the presence of a gradually increasing Δ[oxCCO] response with increasing source-detector separation, neither Δ[Hbdiff] nor Δ[HbT] showed the same consistent behaviour, fortifying the argument that Δ[oxCCO] has stronger brain-specificity than the haemoglobin signals. Interestingly, the Δ[Hbdiff] and Δ[HbT] signals show inconsistent brain specificity between them and between challenges. Although it is not the focus of this paper, this finding warrants further investigation as it has been noted recently that changes in the haemodynamics of scalp cutaneous vessels due to systemic artefacts in evoked experiments are (i) more pronounced in the NIRS Δ[HbO_2_] measurements and (ii) can be different across scalp locations (Kirilina et al., 2012).

Only Δ[Hbdiff] in hypoxia and Δ[HbT] in hypocapnia showed some difference between different detectors, with the magnitude of the response however increasing with *decreasing* source-detector spacing in both cases, a behaviour suggestive of larger extracranial than intracranial hemodynamic changes taking place. A physiological explanation for this finding may rest in the fact that during global challenges for the manipulation of oxygen delivery, both the cutaneous and cerebral vascular beds are affected. The response of skin to hypoxia (Simmons et al., 2007) and hypocapnia (Fujii et al., 2012) is complex and poorly characterised, but it is not unlikely that the differential CO_2_ and O_2_ vasoreactivity curves of the two vascular beds could explain our observations.

### Potential confounding factors

We believe that it is unlikely that the gradient response illustrated in Fig. 3 could have been artificially introduced through the use of different DPFs between detectors, as described in the Differential spectroscopy section in Methods. Reverting back to the individual data sets from which the group data were derived, we investigated whether the behaviour of increasing Δ[oxCCO] amplitude with increasing source-detector spacing would change when the NIRS-derived CCO signal was presented not as Δ[oxCCO], in μmolar, but in μmolar · cm, derived by multiplying the Δ[oxCCO] signal of each detector with the corresponding pathlength. We found that such was not the case and the gradient response in the NIRS-derived and pathlength-independent CCO signal remained unaffected.

The wavelength range that yields the ideal separation of the Δ[HbO_2_] and Δ[HHb] signals is not necessarily optimal for resolving the Δ[oxCCO] signal. Since it was the latter signal that was the primary focus of the current work, it was also the one that guided our selection of wavelengths based on the (limited) literature. Apart from the general consensus that a wavelength range that includes the 830 nm feature of CCO needs to be used, there is no conclusive evidence as to the optimal lower bound of this range for avoiding haemoglobin cross-talk (Heekeren et al., 1999; Kolyva et al., 2012; Matcher et al., 1995). We chose to keep this range fairly tight and start from 780 nm, in order to avoid any distortion introduced by the prominent 760 nm HHb peak to the fitting algorithms. This wavelength range is in agreement with previously published studies from our group and allows for comparability (Tachtsidis et al., 2009; Tisdall et al., 2007, 2008a).

Furthermore, a different wavelength range of the data was used for differential spectroscopy (780–900 nm) and SRS (740–900 nm). Combining attenuation measurements from four detectors and calculating attenuation slopes, renders SRS inherently noisier as a technique than differential spectroscopy. As a result, when SRS was applied to the 780–900 nm portion of the data, we often encountered unrealistic TOI values (> 100%, negative or well outside the ‘normal’ range of 60–75%). With the exception of 1 subject, this problem was overcome by simply expanding the wavelength range at which SRS was performed. On the other hand, a previous study with the same system in healthy volunteers has suggested no effect on the concentration changes derived with differential spectroscopy, of the wavelength range selected for fitting the attenuation data (740–900 nm or 780–900 nm) (Kolyva et al., 2012). We therefore considered it unnecessary in the present study to repeat the differential spectroscopy part of the analysis for the same wavelength range that was eventually used for deriving TOI.

The pHOS system has been specifically optimised for monitoring Δ[oxCCO], by combining measurements of light absorption and scattering at discrete wavelengths with multi-distance measurements of BB light attenuation. The possible interference of changes in optical scattering on a methodology that is based on the assumption that all measured attenuation changes are due to absorption only, and questions about whether the Δ[oxCCO] signal truly arises from CCO and is not simply an artefact of poor chromophore separation by the algorithm used to convert optical density into concentration changes, are the most notable challenges in monitoring Δ[oxCCO] (Cooper and Springett, 1997). However, optical scattering was measured with the pHOS and no change was detected during the experimental protocols. Moreover, the use of BB light for resolving chromophores over hundreds of wavelengths gave confidence that the algorithm used to derive concentration changes could provide sufficient chromophore separation, even in the presence of significantly higher haemoglobin concentrations than CCO.

An effect of haemoglobin cross-talk was not evident in our data. Spectroscopic cross-talk can result from the wavelength dependency of the partial photon pathlength of the activated cortical volume and can lead to erroneous concentration calculations, that are demonstrated as a change in one chromophore mimicking the change in another (Uludag et al., 2004). It becomes immediately obvious from a comparative inspection of Figs. 3 and 4 that the directional response of Δ[oxCCO] triggered by the four experimental paradigms was independent of that of the haemoglobin signals.

### Future work

In future studies, it would be worthwhile using additional source-detector spacings, longer than 3.5 cm, to investigate whether the response of Δ[oxCCO] eventually plateaus or keeps showing further increase with increasing penetration depth. If light penetration was not sufficient for the direct projection of the technology described in this study at longer source-detector distances, use of a time-resolved system could be considered instead. In fact, we have recently developed a multi-channel, multi-wavelength, time-resolved spectrometer with a supercontinuum light source, which can be used for the non-invasive investigation of multi-depth Δ[oxCCO] measurements. Additionally, the ability of this system for multi-channel measurements, when combined with information from structural MRI scans, could facilitate the study of possible regional differences in the Δ[oxCCO] response in different cortical areas and of the effect that the different thickness of overlying extracerebral tissue might have on these measurements.

In the absence of an alternative multi-depth measure of cerebral metabolism to corroborate and reinforce the NIRS-derived Δ[oxCCO] measurements of the present study, an alternative approach would be through mathematical modelling of the cerebral circulation and metabolism (Banaji et al., 2008), coupled with a model of light transport through a multi-layer structure of, preferably, the actual head and cortical structure of each subject (provided by MRI scans). Such an approach would enable us to derive predictions of the Δ[oxCCO], Δ[HbT] and Δ[Hbdiff] responses at different depths, for the various challenges.

In future studies, skin blood flow measurements could prove to be a useful additional measure for the interpretation of the NIRS haemoglobin signals. As explained in the Source-detector spacing and depth-dependence of NIRS signals section, some degree of extracranial contribution is expected to be present in the recordings from all detectors, and a modality providing independent information specifically about scalp perfusion changes could prove to be helpful in uncoupling the extra- and intracerebral physiological mechanisms that contribute to the haemoglobin changes measured with NIRS during the various challenges. Additionally, Monte Carlo simulations using the model described above could provide valuable insights on the effect of individual extracerebral anatomy on the photon travel.

Finally, the investigation of the temporal correlations or dissociations between the NIRS parameters and the co-registered hemodynamic data could provide significant added knowledge on the physiological mechanisms triggered during the challenges and is going to be the focus of further work.

## Conclusions

We have shown that the magnitude of the Δ[oxCCO] response to changes in cerebral oxygen delivery increases with light penetration depth, possibly reflecting the higher intracerebral tissue concentration of CCO. Such a depth-dependent behaviour was not observed in the haemoglobin signals. This finding is indicative of the superior brain-specificity of Δ[oxCCO] and fortifies its clinical appeal as a non-invasive marker of regional cerebral metabolic status.

The following is the supplementary data related to this article.

Supplement Fig. 1 Group data of the time course of mean flow velocity in the middle cerebral artery (Vmca), for hypoxia (A), hyperoxia (B), hypocapnia (C) and hypercapnia (D). The corresponding traces of SpO_2_ (A), end-tidal partial O_2_ pressure (B) and end-tidal partial CO_2_ pressure (C and D) are also provided for reference. The small symbols on top of each plot indicate statistical significance with respect to time point 1 (P < 0.05) and the error bars represent the standard error of the mean.

## Figures and Tables

**Table 1:** Patient demographics and systemic variables.

	Hypoxia		Hyperoxia		Hypocapnia		Hypercapnia	
n	15		15		15		15	
Age	30 ± 4 years		30 ± 4 years		31 ± 4 years		30 ± 3 years	
Gender	10 male		10 male		9 male		11 male	

	Point 1	Point 9	Point 1	Point 9	Point 1	Point 9	Point 1	Point 9

MBP	84.2 ± 1.6	88.1 ± 4.3	88.7 ± 3.3	88.7 ± 3.3	91.3 ± 4.7	96.8 ± 7.2	90.2 ± 4.3	105.6 ± 4.2⁎
HR	72.6 ± 3.4	88.6 ± 4.1⁎	74.2 ± 3.1	70.5 ± 2.5⁎	75.3 ± 2.3	91.0 ± 2.2⁎	72.5 ± 3.0	81.5 ± 3.4⁎
SpO_2_	97.4 ± 0.6	80.0 ± 1.3⁎	97.2 ± 0.5	98.2 ± 0.6⁎	97.4 ± 0.5	97.3 ± 1.1	97.7 ± 0.5	97.4 ± 0.5
EtCO_2_	5.5 ± 0.2	5.2 ± 0.1⁎	5.5 ± 0.1	5.1 ± 0.1⁎	5.3 ± 0.2	3.0 ± 0.1⁎	5.3 ± 0.1	7.7 ± 0.2⁎
Vmca	0	14.3 ± 3.7⁎	0	− 3.5 ± 2.3	0	−30.0 ± 2.3⁎	0	59.1 ± 6.0⁎

MBP: Mean blood pressure (mmHg); HR: Heart rate (beats per minute); SpO_2_: Arterial oxygen saturation (%); EtCO_2_: End-tidal partial pressure of carbon dioxide (kPa); Vmca: Flow velocity in the middle cerebral artery (% change from Point 1). ‘*Point 1*’ corresponds to baseline and ‘*Point 9*’ to the end of the challenge. Table entries are mean ± SEM.

⁎ P < 0.05 compared to Point 1.

**Table 2:** Average pathlength.

	Det_3.5	Det_3.0	Det_2.5	Det_2.0
Hypoxia	26.08 ± 4.16	22.35 ± 3.57	15.65	12.52
Hyperoxia	24.42 ± 3.63	20.93 ± 3.11	15.65	12.52
Hypocapnia	24.26 ± 3.37	20.80 ± 2.89	15.65	12.52
Hypercapnia	25.08 ± 3.35	21.50 ± 2.87	15.65	12.52

All values are in cm and are averages over 15 volunteers. For det_3.5 and det_3.0 the FD measurements were used for the assessment of DPF, whereas for det_2.5 and det_2.0 constant DPF of 6.26 was used.

**Table 3:** Total scores of ‘brain specificity’.

	Δ[oxCCO]	Δ[Hbdiff]	Δ[HbT]
Hypoxia	85%	17%	67%
Hyperoxia	81%	33%	67%
Hypocapnia	93%	63%	8%
Hypercapnia	94%	48%	30%
