# Supplementary material for: Cytochrome c oxidase response to changes in cerebral oxygen delivery in the adult brain shows higher brain-specificity than haemoglobin
Source: Neuroimage. 2014 Jan 15;85(Pt 1):234–44. doi: 10.1016/j.neuroimage.2013.05.070 (PMC3898943; doi:10.1016/j.neuroimage.2013.05.070)
Supplement: Supplement Fig. 1 — Group data of the time course of mean flow velocity in the middle cerebral artery (Vmca), for hypoxia (A), hyperoxia (B), hypocapnia (C) and hypercapnia (D). The corresponding traces of SpO2 (A), end-tidal partial O2 pressure (B) and end-tidal partial CO2 pressure (C and D) are also provided for reference. The small symbols on top of each plot indicate statistical significance with respect to time point 1 (P < 0.05) and the error bars represent the standard error of the mean. [file mmc1.docx]

**Supplement Figure 1**

**Supplement Figure 1**: Group data of the time course of mean flow velocity in the middle cerebral artery (Vmca), for hypoxia (A), hyperoxia (B), hypocapnia (C) and hypercapnia (D). The corresponding traces of SpO_2_ (A), end-tidal partial O_2_ pressure (B) and end-tidal partial CO_2_ pressure (C and D) are also provided for reference. The small symbols on top of each plot indicate statistical significance with respect to time point 1 (P<0.05) and the error bars represent the standard error of the mean.
